# Discovery of an unusually high number of de novo mutations in sperm of older men using duplex sequencing

**DOI:** 10.1101/gr.275695.121

**Published:** 2022-03

**Authors:** Renato Salazar, Barbara Arbeithuber, Maja Ivankovic, Monika Heinzl, Sofia Moura, Ingrid Hartl, Theresa Mair, Angelika Lahnsteiner, Thomas Ebner, Omar Shebl, Johannes Pröll, Irene Tiemann-Boege

**Affiliations:** 1 Institute of Biophysics, Johannes Kepler University, Linz, Austria 4020;; 2 Department of Gynecology, Obstetrics and Gynecological Endocrinology, Kepler University Hospital, Linz, Austria 4020;; 3 Center for Medical Research, Faculty of Medicine, Johannes Kepler University, Linz, Austria 4020

## Abstract

De novo mutations (DNMs) are important players in heritable diseases and evolution. Of particular interest are highly recurrent DNMs associated with congenital disorders that have been described as selfish mutations expanding in the male germline, thus becoming more frequent with age. Here, we have adapted duplex sequencing (DS), an ultradeep sequencing method that renders sequence information on both DNA strands; thus, one mutation can be reliably called in millions of sequenced bases. With DS, we examined ∼4.5 kb of the *FGFR3* coding region in sperm DNA from older and younger donors. We identified sites with variant allele frequencies (VAFs) of 10^−4^ to 10^−5^, with an overall mutation frequency of the region of ∼6 × 10^−7^. Some of the substitutions are recurrent and are found at a higher VAF in older donors than in younger ones or are found exclusively in older donors. Also, older donors harbor more mutations associated with congenital disorders. Other mutations are present in both age groups, suggesting that these might result from a different mechanism (e.g., postzygotic mosaicism). We also observe that independent of age, the frequency and deleteriousness of the mutational spectra are more similar to COSMIC than to gnomAD variants. Our approach is an important strategy to identify mutations that could be associated with a gain of function of the receptor tyrosine kinase activity, with unexplored consequences in a society with delayed fatherhood.

There are certain de novo mutations (DNMs) that are highly recurrent, with mutation rates orders of magnitude higher than the genome average. These mutations have been discovered because of their association with congenital disorders. Moreover, these mutations have several other associated characteristics (for reviews, see Arnheim and Calabrese 2009, 2016; Goriely et al. 2009; Goriely and Wilkie 2012): They encode missense substitutions with gain-of-function properties (activating mutations); they occur exclusively in the male germline; and older men have a higher probability of having an affected child than do younger males, known as the paternal age effect (PAE) and described decades ago (Risch et al. 1987; Crow 2000, 2012). In the literature, these mutations have been termed as PAE mutations; selfish mutations; or recurrent, autosomal dominant, male-biased, and paternal age effect (RAMP) mutations, as reviewed previously (Arnheim and Calabrese 2009, 2016; Goriely and Wilkie 2012).

In the past few decades, it was shown that the high incidence levels and steep increase with age of these PAE mutations are not solely owing to errors occurring during the continuous replicative process of spermatogenesis (Risch et al. 1987; Crow 2000, 2012). Instead, it was suggested that these behave like driver mutations, well known in cancer, which are mutations that promote their own clonal expansion. In particular, PAE mutations have been described in genes such as *FGFR2*, *FGFR3*, *HRAS*, *PTPN11*, *KRAS*, and *RET* (Qin et al. 2007; Choi et al. 2012; Shinde et al. 2013; Maher et al. 2016) and more recently in six new genes (*BRAF*, *CBL*, *MAPK1*, *MAPK2*, *RAF1*, and *SOS1*) (Maher et al. 2018), all acting in the receptor tyrosine kinase (RTK)-RAS signaling pathway and expressed in spermatogonial stem cells (SSCs). The mutations modify the signal modulation of the RTK-RAS pathway by an activating effect of the mutant protein. This dysregulation of the RTK-RAS pathway drives the preferential expansion of mutant SSCs, observed in the testis as mutant clusters that become larger with age (Qin et al. 2007; Choi et al. 2008, 2012; Shinde et al. 2013; Yoon et al. 2013; Maher et al. 2016, 2018). Therefore, as men age, the germline becomes a mosaic for multiple PAE mutations, all in different anatomical locations of the testes (Shinde et al. 2013; Arnheim and Calabrese 2016; Maher et al. 2018), suggesting that each mutation arises and expands independently.

The clonal expansion of some PAE mutations in the testis with age also results in more mutant sperm in older individuals and an increased incidence of the associated congenital disorder with paternal age, as observed, for example, for achondroplasia (ACH), a growth disorder with a defect in FGFR3 signaling (Risch et al. 1987; Tiemann-Boege et al. 2002; Shinde et al. 2013). The transmission of mutations in SSCs to sperm and hence to the offspring does not always correlate positively; especially, mutations associated with tumors hardly overlap with spontaneous germline mutations (Goriely et al. 2009; Aoki and Matsubara 2013; Giannoulatou et al. 2013).

In spite of the importance of PAE mutations in the male germline, which is highlighted by their high incidence, increased frequency with paternal age, and phenotypes potentially leading to early or late-onset disorders in children of older men, we know very little about this mutagenic mechanism. There are still many open questions on how and to what extent specific PAE mutations affect cell growth and SSC differentiation and of the role of apoptosis or cell-death counterbalancing clonally expanding cells. So far, studies have focused on well-characterized mutations associated with a disorder (for reviews, see Arnheim and Calabrese 2009, 2016; Goriely et al. 2009; Goriely and Wilkie 2012) or on the most prevalent mutations observed at late oncogenic stages in somatic tumors, which were shown to be unrelated to the selfish expansion of mutant SSCs (Goriely et al. 2009; Giannoulatou et al. 2013; Maher et al. 2016). However, many genes in the RTK-RAS signaling pathway, or in other pathways, could harbor as-yet-unknown mutations that could be expanding with paternal age but have gone undetected so far, although they may have important health consequences in children of older fathers. To overcome this, a more recent study used a sequencing strategy to discover prospective driver mutations in genes of the RTK-RAS pathway by enriching for mutant SSCs identified in testes biopsies by a high RTK activity that identified 61 mutations at a frequency of ≥0.06% (Maher et al. 2018).

In this work, we further developed the discovery of prospective RTK mutations in the male germline to include ultra-low-frequency variants (with a VAF of <2 × 10^−5^). For this purpose, we used sperm because this biological material is easy to sample and provides information on whether mutations are passed on throughout the different maturation and differentiated stages of the male germline. Accurately identifying ultra-low-frequency mutations by sequencing is still technically very challenging, especially if no enrichment of mutants is possible (e.g., high RTK signaling). To date, only a few methods can achieve the required sensitivity and accuracy. One of these methods is duplex sequencing (DS), a strategy that organizes sequence reads derived from the same DNA molecule into families with information on the forward and reverse strands (Schmitt et al. 2012). Given the excessive sequencing depth required in DS, in this work we focused on the coding region of *FGFR3* because (1) this gene has well-characterized PAE mutations; (2) various reported DNMs associated with congenital disorders have incidence levels that fall within the sensitivity of DS (for reviews, see Goriely et al. 2009; Goriely and Wilkie 2012; Shinde et al. 2013; Maher et al. 2018); and (3) *FGFR3* has been categorized as an oncogene with many missense mutations and high oncogenic score (Tomasetti and Vogelstein 2015).

## Results

### DS detects ultra-low-mutation frequencies

**Figure 1.**
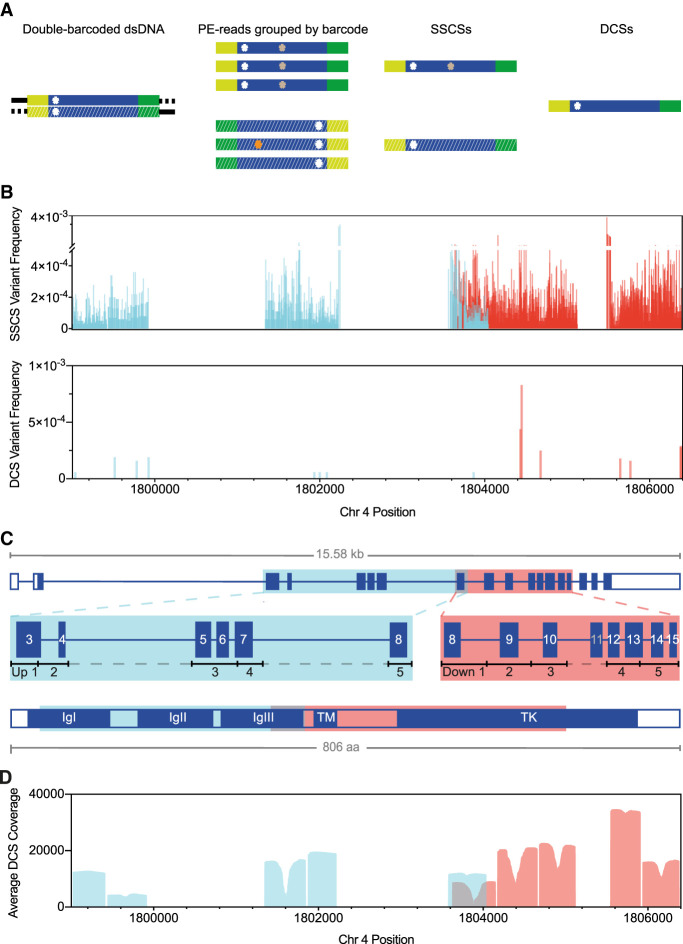
DS using *FGFR3* targeted sequencing strategy with enzymatic digestion. (*A*) DS overview: Barcoded adapters (yellow and green) were ligated at both ends of size-selected restriction enzyme–digested genomic fragments (blue). After several rounds of PCR amplification and hybridization captures, libraries were sequenced on the Illumina MiSeq (v3 600-cycles); for sequence analysis, paired-end (PE) reads were grouped into SSCSs, and complementary SSCS were joined into a DCS. Only DCS with mutations in both complementary SSCS (white asterisk) were considered true variants. Colored asterisks represent artifacts (e.g., sequencing and PCR errors). (*B*) Variants detected at 2881 different positions of the total 4405 sequenced positions identified in SSCSs (*n* = 14,552 total variants; VAF = 1.0 × 10^−5^ to 3.8 × 10^−3^) or at 15 positions in DCSs (*n* = 15 total variants; VAF = 6.0 × 10^−5^ to 8.3 × 10^−4^) of two *FGFR3* libraries (*FGFR3* Up O Oct19 Re-seq and *FGFR3* Down O BAT), each targeting either the Up 1-5 (blue) or the Down 1-5 (red) regions/subregions. (*C*) Exonic structure of *FGFR3*. Shown are the five upstream (blue) or downstream (red) regions targeted via restriction enzyme fragmentation that include exons 3 to 8 or 8 to 15 (except exon 11), respectively; restriction digests rendered fragments ∼370–550 bp in size. Acronyms of the FGFR3 structure are as follows: (IgI-III) immunoglobulin-like domain I-III, (TM) transmembrane domain, (TK) tyrosine kinase domain, and (aa) amino acids. (*D*) Average DCS coverage per position of all sequenced libraries.

High-throughput sequencing is a widely used method for prospective screening of mutations, but given its high error rate of ∼0.1%–2%, it cannot be used for detecting low or ultra-low-frequency variants or mutations (for review, see Salk et al. 2018). The sequencing method with the lowest reported error rate using such a tagging approach is DS, which uses a double barcode strategy to retrieve information from both strands of the original DNA molecule. In DS, reads are first organized into families of either the forward or the reverse strand, known as single-stranded consensus sequence (SSCS). Then, the complement SSCS are further grouped into the duplex consensus sequence (DCS) representing both DNA strands of one initial DNA molecule (Fig. 1A). A variant is considered true, if present in the majority of reads of both strands in the DCS, allowing for an extremely low error rate of ∼10^−7^ to 10^−8^, which is a major improvement of at least one to two orders of magnitude compared with other ultradeep sequencing methods (Schmitt et al. 2012; Fox et al. 2014; Kennedy et al. 2014). Most ultradeep sequencing methods use information of only one strand and are limited mainly by DNA lesions (Lou et al. 2013; Arbeithuber et al. 2016). These DNA lesions can be amplified during the library preparation resulting in false positives. DS greatly reduces errors resulting from DNA damage because a true variant is present in both DNA strands, whereas a DNA lesion is only found in one DNA strand (Arbeithuber et al. 2016).

Here, we illustrate the higher accuracy of a DCS over a SSCS, the latter being equivalent to ultradeep sequencing approaches examining only one DNA strand (Fig. 1B). In SSCSs, we observed higher variant allele frequencies (VAF) and many more variants. Most of them were filtered out in the DCSs (Fig. 1B). Note that insertion-deletions were not considered here. Some variants within SSCSs could be PCR jackpots (errors occurring during the initial PCR cycles that are exponentially amplified) (Lou et al. 2013) or, alternatively, the product of DNA lesions as observed previously (Arbeithuber et al. 2016). In fact, the majority (86%) of the called SSCS were C > A transversions (the product of oxo-G lesions) or G > A/C > T transitions (the product of cytosine deamination) as shown in Supplemental Figure S1.

The high accuracy of DS is tied with the disadvantage that this approach is very data expensive, and only a fraction of the input molecules ends up in a DCS. For this reason, we only focused on exons 3 to 15 of the *FGFR3* gene (∼4.5 kb). As shown in Figure 1C, each of our sequencing libraries targeted one of two ∼2.5-kb regions of *FGFR3*. The two regions were divided further into five subregions (Up 1-5 or Down 1-5), each ∼500 bp in size, that could be fully covered by the two paired-end (PE) reads with an Illumina MiSeq v3 600-cycle sequencing kit. Our DS strategy also included a new approach that enriched for these subregions using restriction enzymes followed by the removal of bulk DNA via bead size selection (see Methods). With this DS strategy, we achieved a median DCS coverage of approximately 10,692 (median DCS coverage per library ranges from 6222 to 38,422) (see Fig. 1D; Supplemental Table S1).

To confirm that variants called by our pipeline represent true mutations, six of the mutations identified with DS were measured with other ultrasensitive detection methods, namely, bead emulsion amplification (BEA) as described previously (Shinde et al. 2013) or droplet digital PCR (ddPCR) (see Supplemental Table S2). BEA is an in-house digital PCR method based on the amplification of single molecules on magnetic beads within an emulsion (Boulanger et al. 2012; Shinde et al. 2013). The ddPCR technology (Bio-Rad Laboratories) is based on the partitioning of PCR reactions into thousands of individual reactions within water-in-oil droplets that are read out in an automated droplet flow-cytometer after the PCR reaction is concluded (for further information, see Supplemental Methods) (Hindson et al. 2011). Our DS measurements were similar among methods; albeit, given the low number of events captured in DS, the confidence intervals are larger for DS than for ddPCR. Moreover, in BEA and ddPCR, more starting molecules were used (approximately 300,000 genomes), making these two methods more accurate for the quantification of very low mutation frequencies compared to DS. A Fisher's exact test for all possible comparisons did not reveal any significant difference between the measured mutation frequencies.

**Figure 2.**
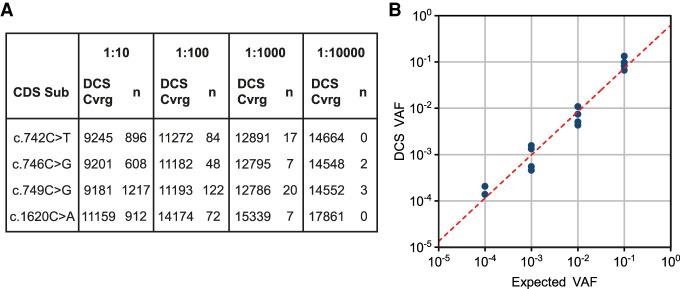
DS spike-in test. (*A*) DNA from four different cell lines (Coriell Cell Repositories; ref: CD00002, NA00711, NA08909, GM18666) was serially diluted into sperm DNA from a young donor in order to prepare four DS libraries (“*FGFR3* Y C 1:10,” “*FGFR3* Y C 1:100,” “*FGFR3* Y C 1:1000,” “*FGFR3* Y C 1:10000”) with expected mutant frequencies of 1:10, 1:100, 1:1000, and 1:10000, respectively. DCS coverage and the number of times the variants were found in each library are shown. (*B*) Expected VAF versus measured VAF with DS; 14 out of the 16 mutations were detected; *R*^2^ = 0.96 (Pearson correlation coefficient).

As a further demonstration of the high sensitivity and accuracy of DS, we performed a mixing experiment. For this purpose, different genomic DNA (gDNA) obtained from Coriell Cell Repositories, each carrying a point mutation (c.742C > T, c.746C > G, c.749C > G, and c.1620C > A), were spiked into DNA from a younger donor at ratios from 1/10 to 1/10,000 in steps of one order of magnitude (Fig. 2A). With this orthogonal assay, we showed that the VAF detected with DS was equivalent to the expected input of the 10-fold dilutions. Note that the starting input amounts varied owing to pipetting errors and the occasional inaccuracy of spectrophotometric measurements used to establish the initial DNA concentration. Fourteen out of 16 variants (four variants per library) were identified. Two replicates of the 1:10,000 dilutions were not detected, likely owing to sampling drop-out at the Poisson distribution level. The Pearson correlation coefficient between observed and expected input dilutions of the 14 measurements was very high (*R*^2^ = 0.96, *P*-value = 9.8 × 10^−10^), showing the power of DS to measure ultrarare variants (Fig. 2B).

### Substitutions detected in the coding region of *FGFR3* in sperm DNA

**Figure 3.**
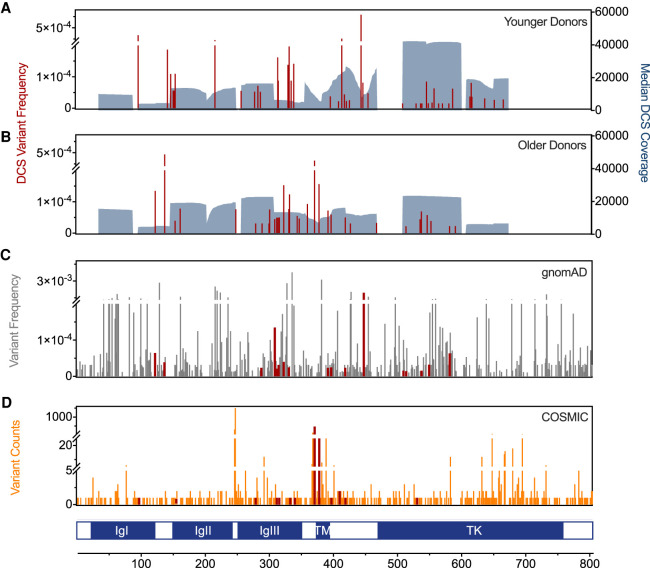
Variant allele frequency (VAF) and DCS coverage. In red are the VAFs of the 99 exonic variant counts found in all libraries from younger (58 counts; *A*) and older donors (41 counts; *B*) listed in detail in Supplemental Table S1. DCS coverage (gray shaded area) is the median DCS coverage at exonic positions of all libraries within the same age group. (*C*) Exonic gnomAD variants for *FGFR3* with frequencies between 1 × 10^−5^ and 1 × 10^−2^. (*D*) Counts of exonic variants associated with tumors (orange) were retrieved from the COSMIC database. The gnomAD and COSMIC variants detected also by DS are shown in red. (IgI-III) Immunoglobulin-like domain I-III, (TM) transmembrane domain, and (TK) tyrosine kinase domain of FGFR3.

In Figure 3, A through D, we show the identified 75 unique variants at 72 different genomic positions in the exonic *FGFR3* sequence (exons 3–15 without exon 11). Of those, 20 have been reported in other databases to be associated with congenital disorders (Human Gene Mutation database [HGMD]) and/or tumors (Catalog Of Somatic Mutations in Cancer [COSMIC]), 13 were unique substitutions associated with tumors (mainly bladder, skin, and multiple myeloma), and 32 were substitutions reported in the Genome Aggregation Database (gnomAD) database (likely viable mutants); 34 DNMs have never been reported before (Fig. 3A–D; Supplemental Table S1). In total, we obtained 12 libraries at an average coverage depth of about 17,000× (up to about 38,000×), each prepared from sperm DNA pooled from five healthy individuals, mainly of European ancestry. Six libraries featured younger donors with ages ranging from 26 to 30 years that captured 58 variant counts (Fig. 3A), and the remaining six featured older donors with ages ranging from 49 to 59 years that captured 41 counts (Fig. 3B; see Supplemental Tables S3, S4). The distribution of donor ages in the young and old pools was significantly different (Supplemental Fig. S2). Further, in spite of differences in the library preparation protocols, we did not observe differences in the overall mutation frequency among libraries, suggesting that our approach is quite robust (Supplemental Fig. S3). Consensus sequences were formed using Du Novo (Stoler et al. 2016, 2020), and variants were called using the Naive Variant Caller (Blankenberg et al. 2014), followed by a more thorough screening with our Variant Analyzer software (Povysil et al. 2021). The Variant Analyzer re-examines raw reads from Du Novo variant calls and provides different summary data that categorize the confidence level of a variant by a tier-based system. This tier classification is based mainly on the number of reads per family and the proportion of the alternative allele in the consensus sequence (see Supplemental Table S5).

**Figure 4.**
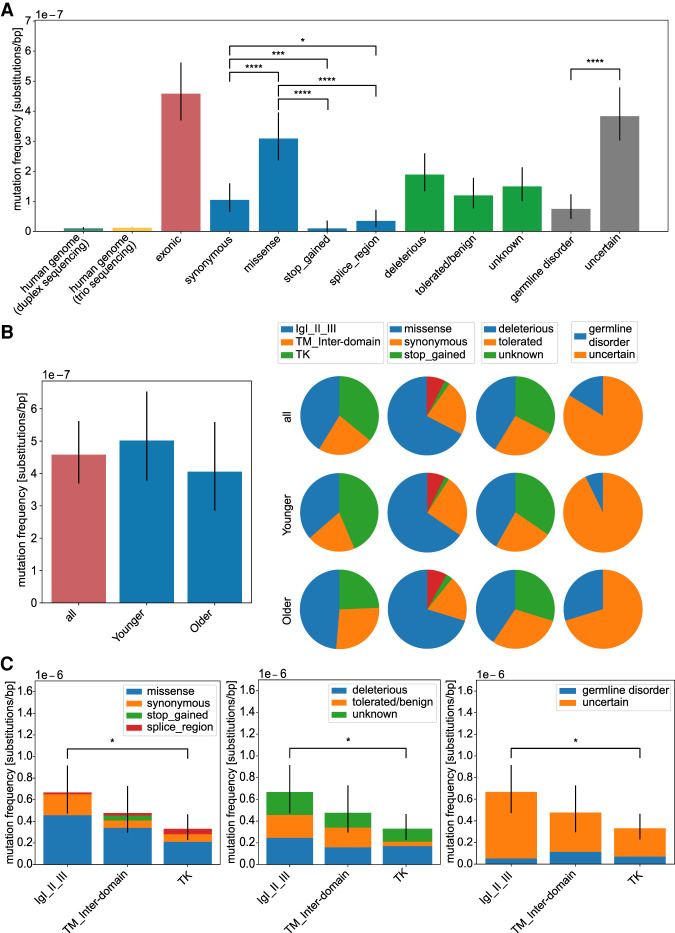
Analysis of mutation frequencies in *FGFR3*. Overall mutation frequencies estimated as number of variants (considered as DNM) divided by the number of sequenced nucleotides of the targeted regions. (*A*) Mutation frequencies (substitutions per base pair) measured in the human genome by duplex sequencing (Abascal et al. 2021) and trio sequencing (Kong et al. 2012) and compared with the exonic regions of *FGFR3* (*n* = 92). We further subdivided the exonic mutations in the type of amino acid substitution (synonymous *n* = 21, missense *n* = 62, stop_gained *n* = 2, splice_region *n* = 7), in the effect on the protein based on the SIFT score (deleterious *n* = 38, tolerated/benign *n* = 24, unknown *n* = 30), or with a phenotype according to the HGMD database (germline disorder *n* = 15, uncertain *n* = 77) listed in detail in Supplemental Table S1. (*B*) Age dependency. Mutation frequencies compared between donor groups (all *n* = 92, younger *n* = 55, older *n* = 37). The pie charts are based on the mutation frequency for each group. (*C*) Domain analysis. Mutation frequencies compared among protein domains (IgI-III *n* = 38, TM_inter-domain *n* = 21, TK *n* = 33) categorized after substitution type, deleteriousness, and associated germline disorder. Error bars are confidence intervals of a Poisson distribution. For pairwise testing, the Chi-square test with Bonferroni–Holm correction was used, and only significant differences are shown in *B* and *C*. (*) *P*-value < 0.05; (****) *P*-value < 0.0001; (n.s.) *P*-value ≥ 0.05.

The mutants detected in this study had a VAF (estimated as the ratio of the alternate allele divided by the reference allele or mutation count per depth of coverage at a given genomic position) between 1.5 × 10^−5^ and 7.9 × 10^−4^ (Fig. 3A; Supplemental Table S1). We also calculated the overall mutation frequency, which is an estimate of the total number of variants per number of sequenced base pairs and can be used to compare overall mutation frequencies with other methods or differences between donor groups or domains (Fig. 4). Note that this mutation frequency or substitution/base pair represents DNM events and was estimated as described in the Methods. As shown in Figure 4A, the mutation frequency of the exonic region of *FGFR3* is about 4.58 × 10^−7^, which is one order of magnitude higher than that measured genome-wide by DS (1 × 10^−8^) (Abascal et al. 2021) or by sequencing trio pedigrees (1.2 × 10^−8^) (Kong et al. 2012). Unlike genome-wide data, *FGFR3* had a particularly higher frequency of transversions and non-CpG transitions (Supplemental Fig. S4), suggesting a unique mutagenic mechanism in this gene explored in more detail in the next sections. The number of missense mutations was significantly higher compared with synonymous, stop-gained, or splice region variants, with ∼65% of the missense variants being deleterious based on the SIFT score (Supplemental Fig. S5). Also, deleterious mutations or mutations with an unknown effect based on the SIFT score were overall more frequent than tolerated/benign variants (Fig. 4A). Note that roughly one-third of the mutations are expected by chance to be synonymous. A proper evaluation of differences between synonymous versus nonsynonymous substitution frequencies can be found in the following section (see *d*_N_/*d*_S_ analysis).

A closer look into the younger and older sperm pools did not show differences in mutation frequencies between these two pools (Fig. 4B), although the mutation frequency in the younger donor pool was slightly higher than in the older donor pool (5.0 × 10^−7^ vs. 4.1 × 10^−7^, respectively). However, some interesting trends can be observed in the older donor pools, such as a higher mutation frequency in the immunoglobulin-like domains I, II, and III (IgI-III) but a lower frequency in the TK domain, as well as a higher frequency of substitutions with a reported germline disorder. These trends suggest that older donors might harbor more substitutions associated with a phenotype that follow a PAE.

### Mutational analysis per domain

Next, we explored if the different domains of *FGFR3* are a repository of DNM. Ninety-two variants (variants occurring twice or more in the same library were counted only once) were distributed at equal numbers across the three main domains (IgI-III *n* = 38, transmembrane (TM) plus flanking inter-domains *n* = 21, and tyrosine kinase [TK] *n* = 33); yet normalized by mutation frequency, the TK domain had the lowest mutation frequency. This domain, however, had the largest proportion of deleterious mutations (Fig. 4C; Supplemental Table S1).

We captured in the TM and surrounding regions the highest mutation frequency of DNM associated with germline disorders (Fig. 4C). Also, the proportion of missense substitutions compared with synonymous substitution was highest in this domain. Additionally, in this domain, we also observed substitutions with the highest VAF (e.g., p.Y373C, p.G380R, and p.E447K); ∼50% higher in magnitude than the median of the other variants (approximately 4 × 10^−5^) measured in *FGFR3*. Two of those variants (p.Y373C and p.G380R) were found only in the older donor pools and have been reported to be associated with congenital disorders such as thanatophoric dysplasia type I (TDI) or ACH. Note that a higher number of COSMIC hits are also observed in this domain (Fig. 3) but not in the general population (gnomAD).

**Figure 5.**
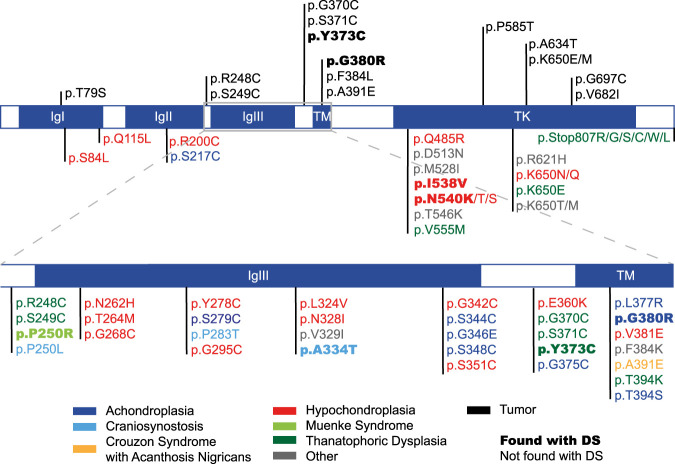
*FGFR3* variants associated with germline disorders and/or tumors. Shown are FGFR3 protein domains and associated variants documented to cause germline disorders (color-coded like the respective disorder) according to the HGMD database and the variants with the most counts in the COSMIC database. Variants captured with our DS approach are highlighted in bold. (IgI-III) Immunoglobulin-like domain I-III, (TM) transmembrane domain, and (TK) tyrosine kinase domain.

Finally, our DS show that the IgI–IgIII domains accumulated a high frequency of missense variants. According to the HGMD database, the IgIII domain harbors a large number of variants associated with congenital disorders (Fig. 5). However, we only detected two out of 20 of these: c.1000G > A (p.A334T) and c.749C > G (p.P250R), which cause craniosynostosis and Muenke syndrome, respectively.

### Mutational mechanism

Aiming to explore the possible mutational mechanisms behind our variants, we tested if the observed variants have a selective advantage linked to the clonal expansion of driver mutations. In evolutionary genetics, the measure of *d*_N_/*d*_S_ (ratio of nonsynonymous vs. synonymous substitutions per nonsynonymous or synonymous sites, respectively) is indicative of positive, neutral, or negative selection. The interpretation of this ratio is that a number close to the reference value (usually one) means no selection; a ratio below the reference means negative selection, and a ratio above the reference implies positive selection (revised by Nielsen 2005). The reference value is calculated by the distribution of *d*_N_/*d*_S_ occurring at random with respect to nonsynonymous versus synonymous sites (1000 iterations). Our analysis of *FGFR3* (Supplemental Table S6) shows that there is a slight increased ratio above neutrality indicative of positive selection when taking all the data together with a *d*_N_/*d*_S_ = 0.71 compared with the reference (median = 0.52), although it lies within the range of neutral expectations (lower range 0.34, upper range 0.75). The strongest indication for positive selection was in the TM domain of the young donor pool with a *d*_N_/*d*_S_ = 1.29, which was significantly higher to the reference (median = 0.71; lower range 0.16, upper range 1.22). Also, of interest was the IgI–IgIII domain in older donors indicating positive selection (*d*_N_/*d*_S_ = 0.79, reference median = 0.48; lower range 0.24, upper range 1.06).

We further analyzed the mutational spectra, transcriptional bias, and mutational signatures in our data (Supplemental Fig. S6A). Based on the cosine similarity of the *FGFR3* mutation spectra, there is a greater similarity with the COSMIC variants than with variants reported in gnomAD (cosine similarity of 0.93 vs. 0.87, respectively) shown in Supplemental Table S7 and Supplemental Figure S7A. This suggests that the type of substitutions in *FGFR3* are more similar to the driver variants reported in tumors for *FGFR3* (COSMIC) compared with random DNMs captured in the general population (gnomAD). Also, note that we do not observe differences in the mutational spectra between the younger and older donor groups (Supplemental Fig. S8), with both groups showing a high cosine similarity score (0.92). The lack of a strong strand bias in the substitution types also indicates that transcription cannot explain our data (Supplemental Fig. S6B). Finally, the mutational signature that compares the variants also in the context of the 5′ and 3′ base adjacent to the variant shows that the main substitutions were strong-to-weak transitions in the context of CpG sites (VCG). Further, the comparison of our observed mutational signature with COSMIC indicates an overlap of ∼51% of the variants explained by pattern SBS24 (aflatoxin exposure), 30% by SBS1 (spontaneous or enzymatic deamination of 5-methylcytosine to thymine), and 18.6% by SBS5 (unknown) (see Supplemental Fig. S9). We have no knowledge if donors had a particular high exposure to aflatoxin. However, it is likely that driver genes have their own mutational signature similar by chance to other reported signatures that needs to be explored further.

### Testis-specific clonal expansion or postzygotic mosaic?

It is possible that the captured variants are expanding clonally with age only in the testis. Alternatively, the high VAF can also be explained by a DNM occurring during postzygotic development, thus affecting a larger number of cells (the earlier in development, the wider might be the range of affected tissues) and, consequently, originating a so-called mosaic state (Rahbari et al. 2016). Especially, mutations in genes of the RTK pathway have been documented as postzygotic mosaics (PZMs) (for review, see Tiemann-Boege et al. 2021). To distinguish between these two possibilities, we further focused on 12 variants (11 amino acid substitutions) that either (1) co-occurred in both the younger and older donor pools (nine amino acid substitutions) or (2) occurred exclusively in older donors (two variants) at frequencies in the upper range (>1 × 10^−4^) (see Table 1). The two latter variants, c.1118A > G and c.1138G > A, with a VAF of approximately 3 × 10^−4^ and approximately 1 × 10^−4^, respectively, were captured in multiple libraries from older donors but not in young libraries. Both variants are also associated with congenital disorders: variant c.1118A > G (p.Y373C) causes TDI, and variant c.1138G > A (p.G380R) is linked to ACH (Fig. 5). The latter has been described as a PAE disorder with the germline of older men carrying more mutations (Tiemann-Boege et al. 2002; Shinde et al. 2013).

**Table 1.**
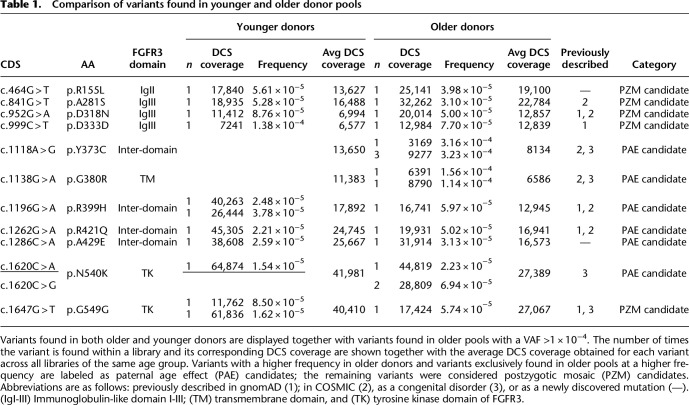
Comparison of variants found in younger and older donor pools

It is possible that variants occurring only in the older donor pool might be testis-exclusive mosaics growing larger over time (see Table 1, PAE candidates). Other interesting variants are c.1620C > A and c.1620C > G. Both are missense mutations that result in the same amino acid substitution (p.N540K) and were found three times in two different older donors libraries (VAF of 2.2 × 10^−5^ and 7.0 × 10^−5^), whereas in younger donors libraries, they were found only once (VAF of 1.6 × 10^−5^). This amino acid substitution is known to be associated with hypochondroplasia, which also suggests that this variant could expand in the male germline with age. An in-depth analysis of more sperm samples or the distribution within testis with a less expensive approach will provide more details in this regard.

**Figure 6.**
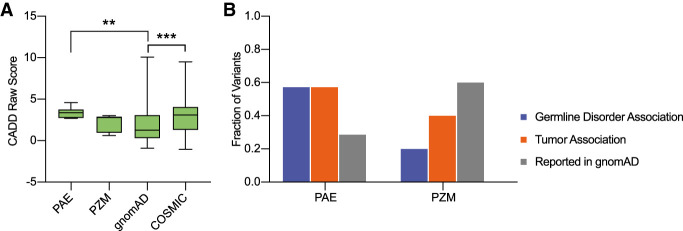
Predictive deleteriousness of variants and association with germline disorders and tumors. (*A*) Four groups of exonic single-nucleotide substitutions were analyzed: mutations selected from all sequenced libraries as a potential paternal age effect (PAE; *n* = 7), mutations selected from all sequenced libraries as a potential postzygotic mosaic (PZM; *n* = 5), exonic single-nucleotide substitutions from the gnomAD v2.1.1 database (gnomAD; *n* = 876), and exonic single-nucleotide substitutions from the COSMIC v90 database (COSMIC; *n* = 397). Boxplot comparison of the CADD raw scores obtained for each variant group. A higher score reflects a higher probability of a deleterious effect. Pairwise testing was performed using a Mann–Whitney *U* test (multiple comparison correction was applied), and only significant differences are marked: (**) *P*-value < 0.01, (***) *P*-value < 0.001. (*B*) Fraction of variants associated with germline disorders according to ClinVar; fraction of variants associated with tumors according to the COSMIC database; and fraction of variants reported in the gnomAD database.

Albeit challenging, PZMs (labeled as PZM candidates in Table 1) might be distinguished from testis-specific mosaics because they occur at a relatively high VAF already in young sperm donors or testis, whereas PAE candidates form no measurable clusters in testis of younger men (Goriely et al. 2003; Qin et al. 2007; Choi et al. 2012; Maher et al. 2018; Tiemann-Boege et al. 2021; revised by Arnheim and Calabrese 2009, 2016). In Table 1, we categorized variants as PAE candidates if detected at a higher frequency or exclusively in older donors, and as PZM candidates if variants co-occurred in both donor pools at similar frequencies. Note that there are some variants found exclusively in younger donors. These could also be PZM candidates missed in the older donor pool owing to sampling. For those selected mutations, we further investigated if we see functional or biological differences between the PZM and PAE candidates (Table 1). Specifically, we compared the predicted deleteriousness annotated for each variant by the combined annotation-dependent depletion (CADD) scores. CADD translates multiple genome annotations into a single score obtained through machine learning used as a measure of deleteriousness of single-nucleotide variants or small insertion-deletions (Rentzsch et al. 2019). As shown in Figure 6A, PAE candidates had higher CADD scores similar to COSMIC variants, implying that some mutations expanding in sperm can be as deleterious as tumor-associated variants. On the other hand, PZM candidates and gnomAD variants had the lowest CADD scores. This suggests that at least part of the PAE candidate substitutions might have a stronger functional impact than most of the variants currently segregating in the population. Whether these are associated with a PAE and might have a deleterious effect, like a congenital disorder or a late-onset disease (e.g., cancer), or instead be a normal phenotype is not known.

Using the same grouping of mutations, we investigated the association with germline disorders, tumors, or gnomAD (Fig. 6B). PAE candidates contained the largest proportion of variants associated with germline disorders and tumors in comparison to PZM candidates, highlighting further differences between these two groups. Variants found in the general population (gnomAD) were associated more often with PZM candidates.

## Discussion

### Targeted DS of a human gene

The establishment of DS opened exciting new possibilities in ultrarare variant detection with an unprecedented sensitivity for a sequencing-based method (Schmitt et al. 2012; Salk et al. 2018). However, the need for consensus sequences for each DNA strand requires the sequencing of multiple PCR replicates, which translates into extremely high costs if the targeted region is large (Kennedy et al. 2014). The most commonly used strategy to target genomic regions for DS is based on consecutive rounds of hybridization captures and enrichment (Schmitt et al. 2015; Krimmel et al. 2016; Loeb et al. 2019; Salk et al. 2019; Shen et al. 2019). Our DS library preparation approach used restriction enzymes to fragment DNA, followed by size selection. Like the CRISPR-Cas9 approach (Nachmanson et al. 2018), this also enables the selection of predetermined target fragments, achieving also a low number of off-targets. Further enrichment of targeted regions is performed by two rounds of hybridization capture followed by PCR, as previously described (Schmitt et al. 2015). This approach rendered an unprecedented DCS coverage obtained for every library. Supplemental Figures S3 and S10 show variation across different libraries, which can be explained by the different parameters used (e.g., input DNA, PCR cycles, sequencing read number). We also observed variable DCS coverage values between different targeted regions within each library that can be attributed to differences in performance of each restriction enzyme, amplification efficiency of each amplicon, and capture oligo efficiency (Supplemental Figure S10). The lower DCS coverage obtained in the central parts of the restriction fragments is originated by trimming low quality read ends. However, with our DS approach, we achieved a median DCS coverage of approximately 11,000, allowing calling mutations at a frequency of at least 1/10,000.

### Mutation accumulation in *FGFR3*

The higher incidence of mutations in *FGFR3* increasing with age in the paternal germline was first described for ACH (c.1138G > A) by screening a panel of sperm donors of different ages (Tiemann-Boege et al. 2002). Another high-resolution study that analyzed the frequency of all nine possible substitutions in the active site of the TK domain p.K650 observed that the mutation occurrence in sperm of this particular codon is dependent on the activating effect of the variant on receptor signaling (Goriely et al. 2009). Several variants in receptor kinase receptors (e.g., *FGFR2* and *FGFR3*) expressed in the male germline have been classified as driver mutations that accumulate over time because the resulting mutated protein confers a selective advantage to the SSCs, leading to a clonal expansion of the mutant. These mutations have reported occurrences of up to 10^−4^ to 10^−5^ in sperm or testis tissue (for reviews, see Arnheim and Calabrese 2009, 2016; Goriely and Wilkie 2012).

Overall, the higher overall DNM mutation frequencies, the elevated VAFs at individual positions, and the trends of *d*_N_/*d*_S_ for positive selection indicate that substitutions captured in *FGFR3* of sperm cannot be explained by a mutagenic mechanism similar to genome-wide DNM but, instead, might be the result of deleterious/activating mutations that promote the clonal expansion of mutant cells. We also observed that the TK and IgI-III domains accumulated a higher number of nonsynonymous substitutions than synonymous substitutions, suggesting that these domains might be subject to stronger clonal expansions of pathogenic variants. It is expected that these clonal expansions lead to larger clusters and a higher VAF in older donors; however, this might not always be the case, because an expansion starting at an early age might not continue throughout the lifetime of an individual because other counteracting processes might take place (e.g., cell death), as hypothesized also for other driver mutations in the male germline such as *RET* (previously known as *MEN2B*) (Yoon et al. 2013). A larger data set of variants and more sequencing data might show the underlying trends with better power.

Further, we were able to detect seven variants (six amino acid substitutions) that are classified as PAE candidates and are likely expanding in the male germline (Table 1). Three out of six of these amino acid substitutions have been associated with PAE disorders and are suspected to increase with paternal age. One of these PAE candidates is c.1118A > G (p.Y373C) measured at a VAF of approximately 3 × 10^−4^, one of the highest frequencies measured in our data set. This variant is causal for the autosomal dominant congenital disorder TDI, also described as a PAE disorder (Rousseau et al. 1996b; Wilcox et al. 1998). Similarly, the c.1138G > A (p.G380R) variant measured at a frequency of approximately 1 × 10^−4^ (very close to the frequency reported in other studies) (Tiemann-Boege et al. 2002) is associated with the autosomal dominant congenital disorder of ACH, another PAE disorder (Rousseau et al. 1994). This mutation is also known to increase in frequency in the sperm of older donors (Tiemann-Boege et al. 2002), and it was reported to confer an advantage to the SSCs and to form clustering in the testis of an older man (Shinde et al. 2013). Another potential driver mutation rendering the amino acid substitution p.N540K was also enriched in the older donor pool. Note that for this missense mutation, we detected two different nucleotide substitutions (c.1620C > A/G). The C > A substitution was found in an older donor library with a VAF of 2.23 × 10^−5^, and the C > G substitution was detected at a VAF of 6.94 × 10^−5^. These substitutions were either lower (1.54 × 10^−5^) or absent in the younger donors pool, respectively. These two variants were also identified in a study measuring prospective mutations enriched in testis biopsies with a high RTK activity (Maher et al. 2018). The p.N540K amino acid substitution is related to the skeletal dysplasia associated with a PAE, the autosomal dominant congenital disorder hypochondroplasia (Bellus et al. 1995; Rousseau et al. 1996a). The higher frequency detected in older donors coupled with an association to a PAE disorder suggests that the p.N540K substitution might be also a testis-specific mosaic expanding with age.

Apart from the few characterized PAE candidates, our study identified further variants that could be potentially expanding with paternal age and have not been described before. These occur mainly within or downstream from the TM domain (e.g., p.T394T, p.R399H, p.R421Q, p.A429E) and are associated with a high deleteriousness and tumors (Fig. 6). Moreover, they are viable because they have been detected in the general population. Thus, our approach is an important tool to identify prospective mutations expanding with paternal age, but more evidence is required to better characterize these trends.

### Testis-exclusive mosaics versus postzygotic mosaicism

Driver mutations and their association with congenital disorders gain a special importance in industrial societies in which parenthood is delayed. The full understanding of driver mutations accumulating in the male germline is key to assess the risks and the impact on future generations. We captured several substitutions with VAF over 1 × 10^−5^. These are viable variants found in the human population (gnomAD), and some of them have also been associated with tumors (COSMIC). Because they were not detected in multiple libraries and/or their frequency was not higher in older donors, we considered that these might be occurring postzygotically, although we did not have further somatic tissue to inspect the presence of the same variant in other tissues of the same donors. The expansion of PZM variants with age is not yet fully understood. In theory, the fitness of these variants conferred to the host cell should dictate their development over time. Therefore, some mosaic variants might expand clonally, and others might remain at low frequencies. But similar to gonadal-exclusive mosaics, these would also be associated with a high recurrence risk in the offspring. Depending on how early in development they occur, the range and expansion might vary (for review, see Tiemann-Boege et al. 2021), but a postzygotic mutation might be widespread and also increase with age (Acuna-Hidalgo et al. 2017; Salk et al. 2019). More importantly, if a DNM occurred before the separation of the somatic and gonadal lineages, then these would be equally likely to be found at high levels in younger sperm donor pools or in more pieces of testis biopsies (for such an example, see Maher et al. 2018). Yet, in blood, the higher number of aberrant clonal expansions observed in older individuals is not fully explained by this theory. In the germline, driver mutations expand clonally with age and its frequency is higher in older individuals, but in the case of postzygotic mutations occurring early in development, this is currently unknown. As reviewed previously (Tiemann-Boege et al. 2021), different lineages of a PZM variant might have distinct fates during development depending on the tissue. Our data suggest that PZM variants are less deleterious (lower CADD score) and are more similar to viable gnomAD variants. Further, PZM variants in the germline might have a “normal” phenotype and are usually not associated with known congenital disorders or tumors.

### Relationship between clonal expansion of mutations and RTK signaling

To date the majority of known PAE mutations affect the RTK or components of its downstream RAS-ERK signaling pathway (*FGFR3*, *HRAS*, *PTPN11*, *KRAS*, *RET*, *RAF1*, *PTPN11*, *BRAF*, *CBL*, *MAPK1*, *MAPK2*) (Tiemann-Boege et al. 2002; Qin et al. 2007; Goriely et al. 2009; Choi et al. 2012; Shinde et al. 2013; Maher et al. 2016, 2018). Dysregulation of the signaling activity of FGFR3 drives the preferential expansion of mutant SSCs, resulting in mutant microclusters in testis observed with DNA-based assays (Qin et al. 2007; Choi et al. 2008, 2012; Shinde et al. 2013; Yoon et al. 2013) or protein markers (Goriely et al. 2003; Giannoulatou et al. 2013; Maher et al. 2016, 2018). The clonal expansion of PAE mutations with age in the testis results in more mutant sperm in older individuals (Tiemann-Boege et al. 2002; Goriely et al. 2009; Maher et al. 2018) and in an increased incidence of the associated congenital disorder.

It has been postulated that the expansion of driver mutations in the testis is proportional to the hyperreactivity of the mutant protein, with larger clusters and more mutant sperm forming with strongly activating mutations compared with mild ones (Goriely et al. 2009; Goriely and Wilkie 2012). However, the transmission of driver mutations to sperm does not always correlate with the in vitro activity of the RTK-RAS signaling pathway by the mutation (Giannoulatou et al. 2013), so there is no simple relationship between the activation of the mutation and the conferred strength of the selective advantage.

Moreover, even though the molecular and cellular events caused by driver mutations in the male germline might parallel the events in tumorigenesis in other somatic tissues, the mutational spectrum observed in tumors hardly overlaps with symptomatic spontaneous germline mutations (Aoki and Matsubara 2013; Giannoulatou et al. 2013). In fact, a study analyzing all possible substitutions in codon p.K650 found that highly activating substitutions associated with cancer (e.g., seborrheic keratoses) were underrepresented in sperm compared with less “oncogenic” substitutions (e.g., p.K650E and p.K650T resulting in thanatophoric dysplasia type II [TDII] and familial acanthosis nigricans, respectively) (Goriely et al. 2009). This difference could be explained by a tissue-specific increase in RTK-RAS signaling pathway activity. Alternatively, the mutations observed in late oncogenic stages in metastatic tissue are usually different from the initial “mild” driver mutations and change during the course of the tumor development (Sottoriva et al. 2015; Tomasetti and Vogelstein 2015). Or yet, very deleterious mutations could be detrimental in development, as it was shown for the most prevalent cancer mutation in *HRAS,* which was diminished in sperm and completely absent in birth data (Giannoulatou et al. 2013).

Accordingly, of the discovered 13 distinct variants associated with tumors, four might be potential PAE candidates. Whether the remaining nine might expand with gonadal age and contribute to viable offspring with a certain disorder or late-onset tumor remains to be tested. These four mutations are located within or nearby the TM domain of the FGFR3 protein, indicating that mutations affecting this particular domain might have a considerable impact on the protein's activity, even in different tissues.

Not only PAE mutations causing early-onset diseases (e.g., RASopathies or skeletal dysplasias) have been described to increase with paternal age (for reviews, see Arnheim and Calabrese 2009, 2016; Goriely et al. 2009; Goriely and Wilkie 2012). Reports have also documented an increased frequency with paternal age of late-onset disorders, like breast cancer or cancers in the nervous system (Hemminki et al. 1999; Hemminki and Kyyrönen 1999) or of neurological and behavioral disorders, including autism (Hultman et al. 2011; Kong et al. 2012; Frans et al. 2013), bipolar disorders (Frans et al. 2008; Grigoroiu-Serbanescu et al. 2012), or schizophrenia (Svensson et al. 2012; for review, see Paul and Robaire 2013; Sharma et al. 2015; Acuna-Hidalgo et al. 2016). Thus, the observed PAE with consequences in neurological, heart, or cancer development might also be partially the result of DNMs in driver genes that lead to abnormalities in the signaling pathway (e.g., RTK-RAS). However, in order to better understand these effects, we need to further investigate DNMs in driver genes in the male germline, how these DNMs affect the self-propagation of the mutation, and the nature of their phenotypic consequences.

## Methods

### Collection of sperm and testes samples

Sperm DNA from anonymous donors mainly of central European ancestry aged between 26 and 30 years and between 49 and 59 years were pooled using five donors per pool; the exact pool composition is shown in Supplemental Table S4. Samples were collected from the Kinderwunsch Klinik, MedCampus IV, Kepler Universitätsklinikum, Linz, following the protocol approved by the ethics commission of Upper Austria (Approval F1-11). Three human gDNA samples encoding the *FGFR3* mutations c.742C > T (NA00711), c.746C > G (NA08909), and c.749C > G (CD00002) were purchased from Coriell Cell Repositories. Similarly, gDNA with the *FGFR3* c.1620C > A transversion (p.540N > K) was extracted from the B lymphocyte cell line purchased from the Coriell Cell Repositories (GM18666).

### Sample and library preparation

Sperm gDNA was extracted using the Gentra Puregene cell kit (Qiagen). In short, DNA of 25 µL of semen was extracted as detailed in the Supplemental Materials and prepared further for the library preparation steps. DS was based on previous protocols (Kennedy et al. 2014) with substantial modifications, including an initial restriction enzyme digest to preselect the target regions. We used in the different libraries some modifications to our main protocol either starting with distinct input DNA amounts, different adapters, or amplification strategies. Supplemental Table S3 summarizes the differences between each of our library preparation protocols. gDNA (500–5000 ng) (Supplemental Table S3) was subject to an overnight restriction enzyme digest that targeted five regions of the *FGFR3* gene (Supplemental Table S8). We used three different adapter synthesis protocols to produce adapter 1, adapter 2, and adapter 3 (see Supplemental Methods; Supplemental Fig. S11).

gDNA was double size-selected (∼300 to ∼600 pb) with SPRIselect beads (Beckman Coulter) and end-repaired and A-tailed using the NEBNext Ultra II end repair/dA-tailing module (New England Biolabs) according to the manufacturer's instructions (see Supplemental Methods; Supplemental Fig. S12). DNA fragments with A-3′ end overhangs were then ligated to the DS adaptors with T-3′ overhangs using the NEBNext Ultra II lLigation module (New England Biolabs), following the manufacturer's instructions, and then purified by 0.8 volumes of AMPure XP beads (Beckman Coulter).

Amplification of ligated DNA was executed using KAPA HiFi HotStart ReadyMix (KAPA Biosystems). Except for four libraries, a strategy of 12 cycles of single primer extensions was adopted before the PCR reaction in order to get multiple copies of the initial genomic template to improve the representation of both forward and reverse SSCS and prevent the exponential amplification of templates resulting in very large family sizes. Reaction volumes and PCR conditions are described in the Supplemental Materials and Supplemental Table S9. Primer sequences and oligonucleotides are shown in Supplemental Tables S10 though S13. After the extension/PCR step, PCR products were purified with AMPure XP beads followed by two to three rounds of targeted capture steps to enrich further for templates of interest. A third targeted capture was performed for two of the libraries using the same procedure as the second one. The number of cycles of both postcapture PCRs varied across libraries (Supplemental Table S3). Pools were verified using fragment analyzer HS NGS (Agilent) as described in more detail in the Supplemental Materials.

Libraries were diluted and pooled for sequencing according to the concentration of dsDNA measured with DeNovix dsDNA high sensitivity. Finally, the libraries were subject to further quantification with the KAPA library quantification kit (KAPA Biosystems). Sequencing reactions were performed on the MiSeq Illumina platform using the kit MiSeq reagent v3 600 cycles (Illumina) at the Center for Medical Research of the Johannes Kepler University and at the VBCF NGS Unit.

### Data processing and variant filtering

FASTQ files were analyzed in Galaxy (on both public [https://usegalaxy.org] and private [zusie.jku.at] servers) according to a DS-specific pipeline that includes an error correction tool (Stoler et al. 2020). Variants (substitutions only) were further inspected and assigned to tiers using the Variant Analyzer (Povysil et al. 2021). Every variant was categorized as a SNP if it was detected in all libraries that shared the same donor pool/target region combination with a frequency ∼10%. Variants with DCS coverage below 1000, intronic variants, and SNPs were discarded from our analysis, and only exonic variants of tier 1 were kept, together with those of tier 2 that were detected more than once (Supplemental Table S14). Selected variants were annotated using Variant Effect Predictor (VEP) (McLaren et al. 2016) and wANNOVAR (Yang and Wang 2015). The VAF was calculated by dividing the number of DCS calling the variant by the DCS coverage at the position of the variant within the library it was detected.

Available data on the *FGFR3* variants were extracted from the gnomAD v2.1.1 (transcript ENST00000440486.2) (Karczewski et al. 2020; https://gnomad.broadinstitute.org) and COSMIC v90 (transcript ENST00000440486.7) (Tate et al. 2019; https://cancer.sanger.ac.uk) databases on January 23, 2020, and January 21, 2020, respectively. Variants from gnomAD were remapped from GRCh37/hg19 to GRCh38/hg38 using the NCBI Genome Remapping Service (https://www.ncbi.nlm.nih.gov/genome/tools/remap). Exonic single-nucleotide substitutions retrieved from gnomAD and COSMIC were annotated with VEP and wANNOVAR. Deleteriousness analysis was performed using CADD raw scores (Rentzsch et al. 2019) extracted from VEP annotation. Pairwise comparison between every group of variants was performed with the pairwise Mann–Whitney *U* test and multiple comparison corrections used a false-discovery rate (FDR) approach (two-stage set-up method of Benjamini, Krieger, and Yekutieli). Germline association was investigated using ClinVar data extracted from wANNOVAR output. Tumor association was investigated by consulting the presence or absence of variants in the extracted COSMIC data.

### Mutation analysis

#### Mutation frequencies versus VAF

The VAF is estimated as the alternate allele count divided by the coverage (reference allele) on a given reference position. The mutation frequency is estimated as the number of different variants per number of sequenced nucleotides (given by the formula below). If the same variant occurred in different libraries, it was counted multiple times; otherwise, it was considered only once:

$$\mathrm{mutation}\;\mathrm{frequency}=\;\frac{\sum_{i}^{\mathrm{n\_libraries}}\mathrm{variant}\;\mathrm{coun}t_{i}}{\sum_{i}^{\mathrm{n\_libraries}}\mathrm{mean\_coverag}e_{i}\times \mathrm{region\_siz}e_{i}}.$$

#### Mutational spectra, cosine similarity, and mutational signature

We categorized the mutational spectra after the (un)transcribed strand and the mutational signature within the trinucleotide context (includes the 5′ and 3′ adjacent nucleotide to the variant) with the tools SigProfilerMatrixGenerator and SigProfilerPlotting (Bergstrom et al. 2019). The mutational signature is also compared with a catalog of signatures within the COSMIC database v3.2 (Alexandrov et al. 2020) by the tool SigProfilerExtractor (Islam et al. 2021). The mutational spectra were estimated using relative counts (variant divided by the total number of variants) or using mutation frequency, both normalized by the substitution type (number of reference alleles/region size); for example,

$$\begin{array}{r} {\mathrm{mutation}\;\mathrm{frequency}}_{\mathrm{substitution}\;\mathrm{type}}=\\ \frac{\sum_{i}^{\mathrm{n\_libraries}}{\mathrm{variant}\;\mathrm{count}}_{i}^{\mathrm{substitution}\;\mathrm{type}}}{\frac{{\mathrm{nt}}_{\mathrm{reference}\;\mathrm{allele}}}{\mathrm{region\_size}}\times \sum_{i}^{\mathrm{n\_libraries}}\mathrm{mean\_coverag}e_{i}\times \mathrm{region\_siz}e_{i}} \end{array}.$$

Mutational spectra are frequently compared by the cosine similarity. The expected cosine similarity for a reference spectrum was calculated by bootstrapping (1000 iterations) randomly sampled *n* mutations of the sample and the original reference spectra, where *n* is the number of mutations in the query spectra (Abascal et al. 2021).

#### The rate of nonsynonymous versus synonymous mutations

We calculated the *d*_N_/*d*_S_ ratio as previously described (Nielsen 2005; Li et al. 2015). The value of *d*_N_ is estimated as the number of nonsynonymous mutations per possible sites producing a nonsynonymous change. The value of *d*_S_ is the number of synonymous mutations per sites producing a synonymous difference. The numbers of nonsynonymous and synonymous sites were calculated using the Nei–Gojobori method (Nei and Gojobori 1986). Under neutrality, the *d*_N_/*d*_S_ ratio is expected to be equal to one; a ratio less than one is suggestive of purifying selection; and a ratio greater than one is suggestive of positive selection. To assess the probability of a random *d*_N_/*d*_S_ ratio, we generated a distribution of *d*_N_/*d*_S_ ratios that would be expected if the observed spectrum of mutational changes was occurring at random with respect to nonsynonymous versus synonymous sites (1000 iterations).

## Data access

The raw sequencing data generated in this study have been submitted to the NCBI BioProject database (https://www.ncbi.nlm.nih.gov/bioproject/) under accession number PRJNA684907.

## Supplementary Material

Supplemental Material
