# Supplementary material for: Discovery of an unusually high number of de novo mutations in sperm of older men using duplex sequencing
Source: Genome Res. 2022 Mar;32(3):499–511. doi: 10.1101/gr.275695.121 (PMC8896467; doi:10.1101/gr.275695.121)
Supplement: Supplemental Material [file supp_32_3_499__DC1.html]

Discovery of an unusually high number of de novo mutations in sperm of older men using duplex sequencing — Supplemental Material 

# Discovery of an unusually high number of de novo mutations in sperm of older men using duplex sequencing

## Supplemental Material

- Supplemental\_Table\_S1.xlsx
- Supplemental\_Table\_S2.xlsx
- Supplemental\_Table\_S3.xlsx
- Supplemental\_Table\_S4.xlsx
- Supplemental\_Table\_S5.xlsx
- Supplemental\_Table\_S6.xlsx
- Supplemental\_Table\_S7.xlsx
- Supplemental\_Table\_S8.xlsx
- Supplemental\_Table\_S9.xlsx
- Supplemental\_Table\_S10.xlsx
- Supplemental\_Table\_S11.xlsx
- Supplemental\_Table\_S12.xlsx
- Supplemental\_Table\_S13.xlsx
- Supplemental\_Table\_S14.xlsx
- Supplemental\_Methods\_and\_Figures.pdf
